# Food consumption pattern in cervical carcinoma patients and controls

**DOI:** 10.4103/0971-5851.60051

**Published:** 2009

**Authors:** Lakshmi Labani, B. Andallu, M. Meera, S. Asthana, L. Satyanarayana

**Affiliations:** *Department of Endocrinology, Nizam Institute of Medical Sciences, Hyderabad, AP, India*; 1 *Department of Home Science, Sri Sathya Sai University, Anantapur, AP, India*; 2 *Institute of Cytology and Preventive Oncology, ICMR, Noida, India*

**Keywords:** *Cervical cancer*, *food consumption*, *low socioeconomic women*

## Abstract

**Background:**

The uterine cervix is the second most common site of cancer among Indian women.Though the human papillomavirus has been demonstrated to be a causative agent for this cancer, a variety of other risk factors are in play, such as sexual and reproductive patterns, socioeconomic, hygienic practices, and diet. The accumulated evidence suggests that cervical cancer is preventable and is highly suitable for primary prevention. The dietary intake of antioxidants and vitamins like vitamin A, carotenoids, vitamin C, folacin and tocopherol is found to have protective effects against cancer of the cervix. Dietary data regarding cervical cancer are still scanty.

**Objective:**

The present study was therefore undertaken to study the dietary pattern among uterine cervical cancer patients and normal controls.

**Materials and Methods:**

A total of 60 consecutive patients and 60 controls were enrolled from a referral hospital during the year 2004. A schedule inclusive of the food frequency pattern and 24-h dietary recall along with the general information was administered to all the enrolled subjects to describe findings on the food consumption pattern along with other important factors.

**Results:**

The mean intake of energy, protein, vitamins, etc., between the cases and controls was not significantly different except for the vitamin C level. Serum vitamin E was found to have lower average in patients as compared to controls. The nutrient intake of cervical cancer patients and controls was grossly deficient in the socioeconomic group studied. With regard to the macronutrient intake, calorie and protein intakes showed a deficit of around 50% when compared to RDA.

**Conclusion:**

The food consumption profile was not significantly different between cervical cancer patients and normal controls.

## INTRODUCTION

Cervical cancer is the second leading malignancy among females in developing countries, including India. The primary underlying cause of the disease is the infection of human papillomavirus (HPV). It usually takes nearly 10–20 years for a precancerous lesion to develop into cancer. Though effective intervention exists, 95% of women in developing countries have never been screened. The factors like the age at marriage, age at consummation of marriage, parity, and history of promiscuity, and the use of oral contraceptives have also proven to be associated with cervical cancer. Dietary patterns have a protective effect against the development of a variety of cancers, particularly those of the epithelial origin.[1] Carotenoids, vitamin A, vitamin C, and folate may reduce the risk of cervical cancer.[2] The increased intake of fruits and vegetables is found to be protective against the incidence of cervical cancer. Low serum carotenoid concentrations may be associated with the risk of developing cervical intraepithelial neoplasia (CIN). The principal nutrients of fruits and vegetables thought to provide protection are antioxidants.[3] Thus, serum carotenoid concentrations may serve as biomarkers of the fruit and vegetable intake. There are limited studies conducted on diet and cervical cancer. Thus, this study attempts to provide data on the food consumption pattern among cervical cancer patients and normal controls.

## MATERIALS AND METHODS

This was a hospital-based descriptive study undertaken in New Delhi to study the dietary pattern of cervical cancer patients and normal controls. The Lok Nayak Hospital was selected for the study. A total of 60 consecutive patients along with an equal number of age-matched controls were enrolled for this study. The patients were selected from the Gynecancer Clinic and Radiotherapy OPD and controls were women without cancerous symptoms attending the general gynecology OPD during the period from April to November of 2004. To estimate different levels of food intake in cases and controls, a sample size of 60 each was found to be adequate.

An interview schedule was formulated and the direct personal interview method was adopted wherein general information pertaining to age, occupation, income, marital status, and education, age at marriage, parity, promiscuity, and type of diet was obtained. Twenty-four-hour dietary recall and food frequency methods were used to obtain the information regarding their food intake pattern.

Biochemical analysis of the serum was done only on a subsample of cases (*n* = 10) and controls (*n* = 10) due to constraints in the study. The analysis of vitamin C and vitamin E was done by dinitrophenyl hydrazine method[4] and dipyridyl method,[5] respectively.

### Statistical methods

The data were coded and were entered into SPSS (Statistical Package for Social Sciences), version 10.0, for analysis. The intake of various foods and other variables between cases and controls were compared with parametric and nonparametric tests for independent groups wherever appropriate.

## RESULTS

Of the total 60 cervical cancer women enrolled, 70% were undergoing radiotherapy treatment while others were with a combination of surgery and chemotherapy. The clinical staging of the selected cases was as follows: 11.7% were with stage Ib, 28.4% with IIa or b while half of the patients were in stage IIIa or b (50%), and 5% were in stage IVa. The rest of the 5% patients belonged to the postoperative group. Sociodemographic factors studied, like the age of the patient, marital status, educational status, income group, and religion are given in Table 1. There was no significant difference between cases and controls with respect to age, marital status, and education. Most of the subjects (98.3%) in both cases and controls were from a lower or poor income group.

**Table 1:** Sociodemographic factors responsible for cervical cancer

Sociodemographic factors	Patients % (*n* = 60)	Controls % (*n* = 60)	*P*-value
Age at study (years)			
25–35	15 (9)	11.7 (7)	N.S.
36–45	33.3 (20)	45 (27)	
46–55	33.3 (20)	31.6 (19)	
56–65	18.4 (11)	11.7 (7)	
Marital status			
Single	0.0 (0)	0.0 (0)	N.S.
Married	86.7 (52)	76.7 (46)	
Divorced/widowed	13.3 (8)	23.3 (14)	
Educational status			
Illiterate	73.3 (44)	70.0 (42)	N.S.
Primary level	16.7 (10)	13.3 (8)	
Secondary level	6.7 (4)	13.3 (8)	
Graduate	1.7 (1)	3.3 (2)	
Postgraduate	1.7 (1)	0.0 (0)	
Income (per capita)			
Very poor (>300)	8.3 (5)	30.0 (18)	0.002
Poor (300–1,000)	60.0 (36)	61.7 (37)	
Low (1000–2,000)	30.0 (18)	6.7 (4)	
Medium (2,000–3,000)	1.7 (1)	1.7 (1)	
Religion			
Hindu	95.0 (57)	40.0 (24)	<0.001
Muslim	5 (3)	51.7 (31)	
Sikh	0.0 (0)	5.0 (3)	
Others	0.0 (0)	3.3 (2)	

N.S.: Not significant

Sexual and reproductive patterns studied, like age at marriage, age at consummation of marriage, age at first childbirth, parity, and history of promiscuity are shown in Table 2. Significant differences between cases and controls for ages at marriage, first sexual intercourse, parity, menstrual history, and promiscuity of the husband were observed.

### Food consumption patterns

**Table 2:** Sexual and reproductive factors for cervical cancer cases and controls

Sexual and reproductive factors	Patients (*n* = 60)	Controls (*n* = 60)	*P*- value
Age (years)	47.2 (10.3)	45.8 (9.2)	N.S.
Age at marriage (years)	14.4 (3.7)	16.7 (4.2)	0.001
Age at first sexual intercourse (years)	16.2 (1.9)	17.5 (3.4)	0.001
Age at first child birth (years)	19.4 (3.7)	20.5 (4.0)	N.S.
Parity			
0–3	23.3% (14)	41.6% (25)	0.03
>3	76.5% (46)	58.3% (35)	
Menstrual history			
Regular	10% (6)	30% (18)	<0.001
Irregular	21.7% (13)	40% (24)	
Menopause	68.3% (41)	30% (18)	
Hygiene pattern (mat. used)			
Sanitary napkins	0% (0)	1.7% (1)	N.S.
Homemade cloth pads	31.7% (19)	48.3% (29)	
N.A.	68.3% (41)	50% (30)	
Polygamous husband			
Yes	16.7% (10)	0% (0)	0.001
No	83.3% (50)	100% (60)	

Mean (SD) values are given in parentheses. N.S.: not significant.

Among the patients, 43.3% were vegetarians; 56.7% were nonvegetarians. In contrast, there were 40% vegetarians and 60% nonvegetarians in the control group. Almost all the patients and control subjects consumed a wheat-based diet daily.

Table 3 reveals the frequency of the consumption of various food items in cases and controls. None of the food items in terms of frequency of consumption is different between case and control groups except for the consumption of milk products (*P* = 0.006). Among the patient subjects 36.7% while 25% among controls consumed pulses daily.

The nutrient intake of cervical cancer patients and controls was grossly deficient with reference to RDAs. With regard to the macronutrient intake, calorie and protein intakes showed a deficit of around 50% when compared to the RDA. However, the fat intake was high. The micronutrient intake was quite low with a deficit value of 1879.4 mg, 17 mg, and 70 μg for β-carotene, vitamin C, and folic acid, respectively, among patients. Table 4 depicts median, minimum, and maximum nutrient levels for cases and controls. None of the levels between cases and controls was significantly different except for vitamin C (*P* = 0.048). The daily consumption of green leafy vegetables, carrots, and pumpkin among patients was only 16.7%, 15%, and 0%, respectively. The frequency of consumption of fruits was 40%.

**Table 3:** Frequency of consumption of various food items in cervical cancer cases and controls

Frequency of consumption			Type		*P*-value
			Case (*n* = 60)	Control (*n* = 60)	
Pulses	Frequent	No.	22	15	N.S.
		%	36.7	25.0	
	Rare	No.	38	45	
		%	63.3	75.0	
Green leafy vegetables	Frequent	No.	10	7	N.S.
		%	16.7	11.7	
	Rare	No.	50	53	
		%	83.3	88.3	
Tomato	Frequent	No.	47	48	N.S.
		%	78.3	80	
	Rare	No.	13	12	
		%	21.6	20	
Carrot	Frequent	No.	9	15	N.S.
		%	15.0	25.0	
	Rare	No.	51	45	
		%	85.0%	75.0%	
Pumpkin	Rare	No.	60	60	-
		%	100.0	100.0	
Other vegetables	Frequent	No.	51	43	N.S.
		%	85.0	71.7	
	Rare	No.	9	17	
		%	15.0	28.3	
Fruits	Frequent	No.	27	19	N.S.
		%	45.0	31.7	
	Rare	No.	33	41	
		%	55.0	68.3	
Milk products	Frequent	No.	36	21	0.006
		%	60.0	35.0	
	Rare	No.	24	39	
Eggs	Frequent	No.	4	1	N.S.
		%	6.7	1.7	
	Rare	No.	56	59	
		%	93.3	98.3	
Meat products	Frequent	No.	6	3	N.S.
		%	10.0	5.0	
	Rare	No.	54	57	
		%	90.0	95.0	

N.S.: not significant.

**Table 4:** Nutrient intake levels in cervical cancer cases and controls

Type	Statistics	Energy	Protein	Fat	Calcium	Fe	Mg
Case	Median	867.2	25.6	25.2	181.1	7.4	174.2
	Minimum	108.9	2.3	0.8	43.5	1.0	21.9
	Maximum	2,434.5	69.9	166.5	1,204.9	21.4	464.0
Control	Median	764.5	22.0	22.2	164.0	7.6	174.7
	Minimum	309.7	10.8	1.8	78.9	2.4	24.8
	Maximum	1,680.1	45.1	61.1	675.7	18.1	385.2
	*P*-value	0.057	0.17	0.056	0.09	0.69	0.56
		**Cu**	**Zn**	**β-carotene**	**Retinol**	**Vit. B_1_**	**Vit. B_2_**
Case	Median	0.9	3.1	183.3	45.0	0.6	0.3
	Minimum	0.1	0.2	19.9	5.0	0.1	0.0
	Maximum	2.3	8.1	3,108.5	794.4	1.8	1.2
Control	Median	0.9	3.4	141.1	35.3	0.6	0.3
	Minimum	0.2	0.3	60.6	15.2	0.1	0.2
	Maximum	37.6	251.9	1,438.5	359.6	1.3	30.7
	*P*-value	0.94	0.68	0.098	0.15	0.55	0.63
		**Vit. B_4_**	**Vit. B6**	**Vit. C**	**Folic acid**	**Vit.B_12_**	**Cysteine**
Case	Median	5.1	0.0	20.3	23.8	0.1	123.3
	Minimum	0.4	0.0	0.0	0.9	0.0	0.0
	Maximum	17.6	0.6	165.7	387.2	1.2	396.5
Control	Median	5.0	0.0	14.8	15.6	0.1	98.1
	Minimum	2.1	0.0	0.4	2.6	0.0	31.5
	Maximum	15.4	0.3	105.9	368.3	0.8	455.0
	*P*-value	0.87	0.37	0.048*	0.095	0.098	0.11

Statistical significance is evaluated by the Mann–Whitney U–test.

* indicates significance.

The daily consumption of tomato showed a higher figure of 78.3%. The consumption of other vegetables such as cruciferous vegetables, beans, tubers, or roots was 85%. The daily consumption of green leafy vegetables was found to be low (16.7%) among patients. This was further low in controls (11.7%), though not significantly different.

### Biochemical analysis

The biochemical analysis [Table 5] shows that the serum vitamin C level was 0.10 mg/dl, which is lower when compared to the normal range (0.2–1.9 mg/dl). There is no significant difference between cases and controls for serum vitamin C levels. Similarly, the normal serum vitamin E levels are in the range of 0.3–1.2 μg/dl. The mean serum vitamin E level in the patients was 0.59 mg/dl, which appeared to be in the normal range. On the other hand, serum vitamin E levels are significantly (*P* < 0.001) lower in cases as compared to controls.

**Table 5:** Mean serum vitamin status in cervical cancer cases and controls

	Parameters	
	Vitamin C (mg/dl)	Vitamin E (mg/dl)
	Case (*n* = 10), control (*n* = 10)	Case (*n* = 10), control (*n* = 10)
Mean ± SD	0.10 ± 0.07, 0.06 ± 0.01	0.59 ± 0.11, 0.76 ± 0.08
Normal range	0.2–1.9	0.3–1.2

## DISCUSSION

This study provides the food consumption pattern of cervical cancer patients undergoing treatment and normal women. As the disease is most prevalent in the low socioeconomic group, majority of patients observed in this study were from low- and poor-income groups. The control selection was also done in the same setup and all the controls got included were almost from the same low socioeconomic group. Food frequency consumption was not significantly different between cases and controls for all items except for the milk products. It was observed that the overall food consumption was very low, especially that of fruits and vegetables in both the cases and controls. It does not give any clue on the role of diet but a reflection of the socioeconomic level. Earlier studies reported that vegetables and to a lesser extent fruits are inversely associated with the risk of cancer.[6] None of the nutrient intake levels were significantly different between cases and controls in the present study except for vitamin C. The levels of vitamin C were higher in cases as compared to controls. This is not in agreement with the literature. This is perhaps due to patients' treatment influence. It was reported that vitamins (vitamin C and carotenoids) could be involved in the protective mechanism of plant foods.[7] ICMR recommends a daily intake of 100 gm of GLV, 40 gm of vegetables, and 30 gm of fruits per person, which was not fulfilled in the present study. Vitamin A and its analogs modulate the growth and differentiation of cancer cells presumably by activating gene transcription via the nucleic retinoic acid receptor (RAR) and α, β, and γ retinoid X receptor (RXR).[8,9]

The chemoprevention effect of retinoids was most likely exerted at the tumor promotion phase of carcinogenesis. Retinoids block tumor promotion by inhibiting proliferation, inducing apoptosis, and including differentiation or a combination of these actions.[10,11]

Vitamin C acts as a potent reducing agent (antioxidant) in several hydroxylation reactions making it capable of reducing compounds like molecular oxygen and nitrates (scavenger). It also inhibits malignant transformation and decreases the chromosomal damage in the cells. Lycopene, a pigment found in tomato, acts as a powerful antioxidant thereby preventing damage to DNA by protecting 2-deoxyguanosine against singlet oxygen damage. It suppresses insulin-like growth factor-1-stimulated cell proliferation. American Association of Cancer Research has described the different effects of lycopene, including the reduction in tumor size.[12]

Serum vitamin C level in both cases and controls of the present study was found to be very low and not significant between groups. This is not in conformity with the literature. A low level of vitamin C when compared to the normal levels is indicative of a lack of protection against increased oxidative stress in the cervical cancer patients. Much experimental data indicate that free radicals have a role in the initiation and promotion of cancer,[13] which involves changes in DNA either as a result of an inherited genetic anomaly or damage to the DNA strand. It is likely that any agent capable of modifying DNA could be carcinogenic. Free radicals fall into this category. Highly invasive cancer cells require a certain level of oxidative stress to maintain a balance between proliferation and apoptosis. These cells generate large amounts of hydrogen peroxide, which is involved in the survival of cancer cells. Antioxidants like vitamin C may suppress the H_2_O_2_ signal molecules, thereby inhibiting cancer cell proliferation.[14]

Serum vitamin E levels were however found to be within the normal range in the present study. The levels were significantly (*P* < 0.001) lower in cases as compared to controls. Studies carried out in different populations demonstrate a decreasing trend in serum levels of α-tocopherol with more advanced cervical lesions.[15,16] The protective effect of α-tocopherol may be mediated through its effect on HPV, a virus strongly implicated in the etiology of cervical cancer.[17,18]

This study showed significant differences between cases and controls for various important sociodemographic, sexual, and reproductive factors. None of the important dietary parameters in this study supported the protective role for the disease. This is perhaps due to the selection of patients undergoing treatment as cases. The inclusion of freshly diagnosed cases would probably have resulted in support of the protection role. The other limitation is that the sample size in the present study may not be adequate to evaluate the role of diet as an independent factor while adjusting in multiple regression setups. The objective is confined to the estimation and comparison of dietary parameters due to the lack of such data. The present study makes available dietary profiles of normal women belonging to the low socioeconomic group along with cancer patients receiving treatment. However, diet which is a reflection of the socioeconomic status may play a surrogate role in the presence of other potential factors for cervical cancer but the present study fails to throw any light perhaps due to limitations of this study.

## CONCLUSION

The nutrient intake of cervical cancer patients and controls was grossly deficient in the socioeconomic group studied. With regard to the macronutrient intake, calorie and protein intakes showed a deficit of around 50% when compared to RDA. The food consumption profile was not significantly different between cervical cancer patients and normal controls.
